# Reductions in plasma and urine mercury concentrations following N,N′bis-(2-mercaptoethyl) isophthalamide (NBMI) therapy: a post hoc analysis of data from a randomized human clinical trial

**DOI:** 10.1007/s10534-023-00560-3

**Published:** 2023-11-21

**Authors:** David A. Geier, Mark R. Geier

**Affiliations:** Research Department, Institute of Chronic Illnesses, Inc, 14 Redgate Ct, Silver Spring, MD 20905 USA

**Keywords:** Chelating agents, Clinical trial, Mercury, Toxicology

## Abstract

Environmental mercury exposure possesses a significant risk to many human populations. At present there are no effective treatments for acute mercury toxicity. A new compound, N,N′bis-(2-mercaptoethyl) isophthalamide (NBMI), a lipophilic chelating agent was created to tightly/irreversibly bind mercury. A post hoc dose-dependent analysis of NBMI therapy was undertaken on data from a randomized controlled NBMI human treatment trial on 36 Ecuadorian gold miners with elevated urinary mercury concentrations. Study subjects were randomly assigned to receive 100 milligram (mg) NBMI/day, 300 mg NBMI/day, or placebo for 14 days. For each study subject daily mg NBMI dose/Kilogram (Kg) bodyweight were determined and plasma and urine mercury concentrations (micrograms (µg)/Liter (L)) on study day 1 (pre-NBMI treatment), 15 (after 14 days of NBMI treatment) and 45 (30 days after NBMI treatment) were correlated with NBMI dosing using the linear regression statistic in SAS. Regression revealed significant inverse correlations between increasing per mg NBMI/Kg bodyweight/day and reduced concentrations of urinary and plasma mercury on study day 15 (reduced by in urine = 18–20 µg/L and plasma = 2 µg/L) and study day 30 (reduced by in urine = 15–20 µg/L and plasma = 4 µg/L) and significant correlations between reductions in mercury concentrations in urine and plasma. Significant 30% reductions in urinary mercury concentrations per mg NBMI/Kg bodyweight/day administered for 14 days were observed. This study supports the dose-dependent ability of NBMI therapy to significantly reduce mercury concentrations, particularly in the urine, in an acutely mercury exposed human population. NBMI therapy should be evaluated in other mercury exposed populations.

## Introduction

Mercury (Hg) is a very toxic element that is a ubiquitous source of environmental toxicity to humans. In recent years, the global community has begun to increasingly respond to the threat of Hg toxicity by developing the Minamata Convention (Eriksen and Perrez 2014). The Minimata Convention is a global effort to protect human health and the environment from the emissions and releases of Hg.

Unfortunately, there are still many human populations at significant risk of suffering Hg intoxication. Further, at present, the options for treating Hg intoxication are limited. The two main compounds utilized for the treatment of Hg intoxication are the chelating agents of 2,3-dimercapto-1-propanesulfonate (DMPS) and dimercaptosuccinic acid (DMSA). In experimental studies, DMPS and DMSA were observed to not form true chelate complexes with Hg (George et al. 2004). DMPS and DMSA are charged molecules, which may limit their ability to cross lipid membranes (i.e., the blood-brain-barrier, cell membranes, etc.), and, hence, reduce their clinical effectiveness (Flora and Pachauri 2010). As a result of the limitations in the effectiveness of DMSA and DMPS to treat Hg intoxication, there is a consensus that new and improved compounds need to be developed (Zalups and Bridges 2012).

Therefore, researchers developed a new compound, N,N′bis-(2-mercaptoethyl) isophthalamide (NBMI). NBMI is lipophilic chelating agent specifically created to tightly and irreversibly bind Hg (Clarke et al. 2012). A phase-IIb randomized controlled human treatment trial utilizing NBMI therapy was initiated among 36 Ecuadorian gold miners with documented elevated levels of Hg exposure (Schutzmeier et al. 2018). The purpose of the present post hoc analysis study was to employ new and different analytical methods to examine data generated from the Ecuadorian study. It is the aim of this study to determine the potential dose-dependent therapeutic efficacy of NBMI therapy to reduce plasma and urinary Hg concentrations.

## Materials and methods

### Study design

The phase-IIb randomized controlled NBMI human treatment trial was previously described in great detail (Schutzmeier et al. 2018). The study was designed as a three-armed, randomized, double-blinded, and placebo controlled trial. Study subjects were screened to only include those with urinary Hg concentrations > 15 micrograms (µg) Hg/Liter (L). Each study subject had plasma and urine Hg samples collected on study day 1 (pre-treatment). Each subject was then administered a treatment regimen for 14 days. The three arms of the study consisted of study subjects being randomly assigned to receive 100 milligram (mg) NBMI/day, 300 mg NBMI/day, or placebo. On study day 15, study subjects, again, had plasma and urine Hg samples collected. Finally, on study day 45 (30 days after cessation of treatment) study subjects had plasma and urine Hg samples collected.

### Study subject examination

For each study subject detailed demographic information was collected. All study subjects were Hispanic males. Ages in years, date of birth in years, fish consumption per week, years working with Hg, the use of smelting Hg-amalgam to recover gold, and handling Hg to extract gold ore, but no smelting, were recorded for each study subject. Plasma and urine Hg samples were analyzed by the qualified laboratory, ALS Scandinavia AB in Lulea, Sweden.

### Determining treatment status

The present post hoc analysis study, unlike the original study, combined study subjects from all treatments groups (placebo, 100 mg NBMI/day, and 300 mg NBMI/day) into one overall study group of 36 persons. In order to determine each study subject’s NBMI treatment status within the combined group, daily doses of NBMI/Kilogram (Kg) bodyweight were determined. These were calculated by dividing the mg NBMI/day each study subject received (placebo = 0 mg NBMI/day, 100 mg NBMI/day, or 300 mg NBMI/day) by the recorded bodyweight in Kg measurement for each study subject prior to starting therapy.

### Statistical methods

Statistical analyses were undertaken using the SAS system for Windows, version 9.4 (Cary, NC, USA). Regression statistical models were utilized in this study. For each regression model constructed, beta (β)-coefficients (with 95% confidence intervals and p-values), standardized estimates, and R^2^ values were determined. The null hypothesis for the regression models was that the slope of the lines would equal zero. A two-sided p-value < 0.05 was considered statistically significant.

Linear regression models were constructed to examine the potential correlation between the treatment status for each study subject (mg NBMI/Kg bodyweight/day) and changes in Hg concentrations in plasma and urine with treatment. The changes measured were determined as the differences in Hg concentrations in plasma and urine samples collected on study days 15 and 45, as compared to study day 1. For each regression model constructed, unadjusted and adjusted models were constructed. The adjusted models examined, what, if any impact, the covariates of the subject’s age in years, date of birth in years, weekly fish consumption, years working with Hg, smelting Hg-amalgam to recover gold, and handling Hg to extract gold from ore, but no smelting had on the correlations observed. Regression models were also constructed to examine the potential correlation between Hg concentrations in urine and plasma from samples collected on study day 1. In addition, potential correlations between changes in Hg concentrations in plasma and urine with treatment were evaluated. The changes measured were determined as the differences in Hg concentrations in plasma and urine samples collected on study days 15 and 45, as compared to study day 1.

## Results

Table 1 summarizes the demographic composition of the 36 study subjects examined in this study. All study subjects were Hispanic males.

**Table 1:** A summary of the demographic status of study subjects examined

Variable	Study subjects (n = 36)
Age in years	
Mean ± std (range)	38.64 ± 10.37 (19 to 59)
Date of birth in years	
Mean ± std (range)	1976 ± 10.30 (1956 to 1996)
Bodyweight in Kg	
Mean ± std (range)	79.22 ± 13.44 (54.5 to 107)
Fish consumption per week	
< 1 time per week	13 (36.11%)
≥ 1 time per week	23 (63.8%9)
Years working with mercury	
Mean ± std (range)	11.75 ± 9.72 (0.5 to 40.0)
Smelting amalgam to recovery gold	
Yes	29 (80.56%)
No	7 (19.44%)
Handling mercury to extract gold from ore, but no smelting	
Yes	13 (36.11%)
No	23 (63.89%)
Daily mg NBMI /Kg dose	
Mean ± std (range)	1.67 ± 1.62 (0.00 to 4.76)
Urinary mercury concentration (µg/L)	
Mean ± std (range)	65.18 ± 80.16 (1.66 to 321)
Plasma mercury concentration (µg/L)	
Mean ± std (range)	14.6 ± 19.63 (1.3 to 95.9)

All study subjects were Hispanic males

*Kg* kilogram, *mg* milligram, *NBMI* N,N′bis-(2-mercaptoethyl) isophthalamide, *std* standard deviation

The demographic data for the study subjects indicates that their ages, dates of birth, and bodyweights were fairly typical for an adult population. The variables measuring potential sources of Hg exposure reveal that most had significant potential sources of ongoing Hg exposure. On average, each study subject had worked for about 12 years with Hg and most (> 80%) employed Hg-amalgam smelting techniques to recover gold. On study day 1, the mean Hg concentration in the urine was about 65 µg Hg/L (range = 1.66 to 321 µg Hg/L) and the mean Hg concentration in the plasma was about 14.6 µg Hg/L (range = 1.3 to 95.9 µg Hg/L). On average, study subjects received daily about 1.7 mg NBMI/Kg bodyweight/day (range = 0.00 to 4.76 mg NBMI/Kg bodyweight/day).

**Fig. 1:**
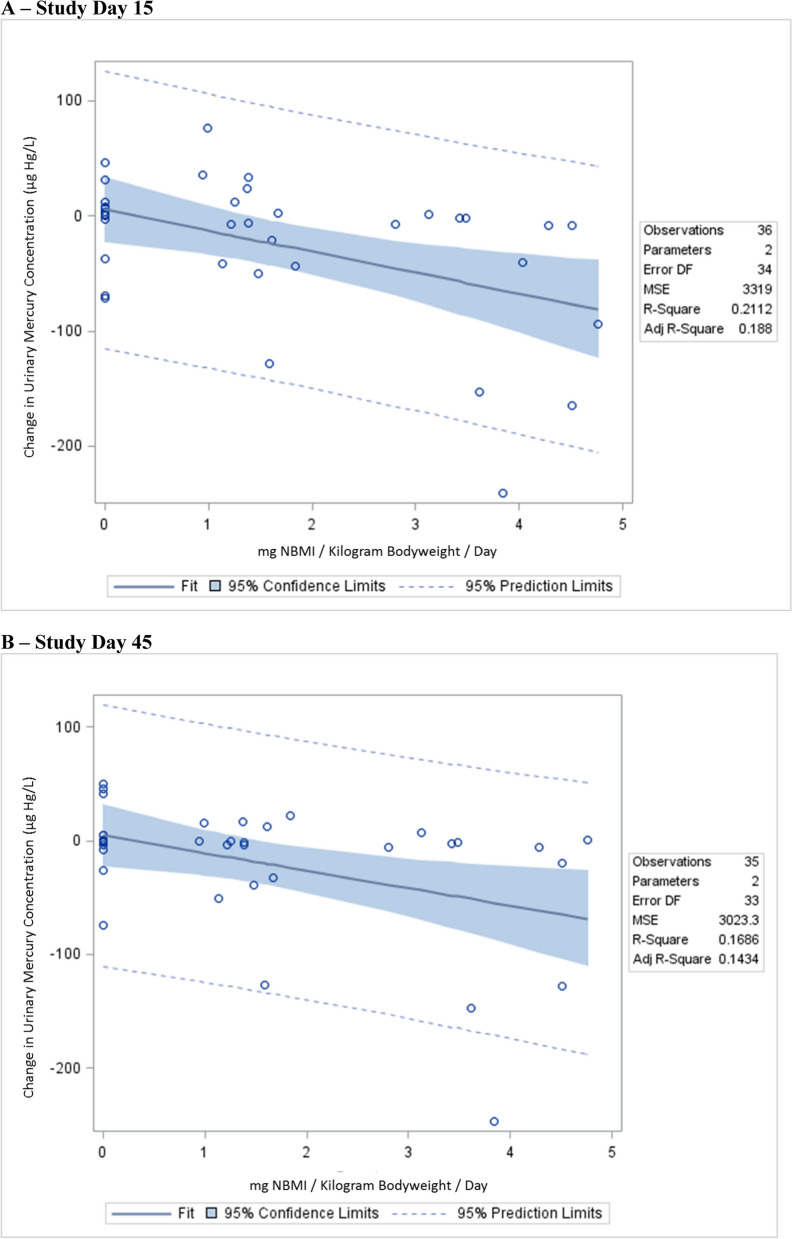
A summary of the correlation between urinary mercury concentrations and daily doses of NBMI.  **A** study day 15,  **B** study day 45

 Table 2; Fig. 1 report the correlation observed between changes in urinary Hg concentrations with NBMI therapy for all study subjects.

**Table 2:** A summary of the linear regression correlation between urinary mercury concentrations and daily doses of NBMI

Study day	Variable	β-coefficient	95% Confidence interval	Standardized estimate	p-value
15	***mg NBMI/Kg/Day***^***1***^	***− 18.13***	***− 30.35 to − 5.92***	***− 0.46***	***0.0048***
15	***mg NBMI/Kg/Day***	***− 20.48***	***− 34.66 to − 6.31***	***− 0.52***	***0.0062***
	Age in years	0.12	− 78.54 to 78.78	0.019	0.99
	Date of birth in years	− 0.49	− 79.62 to 78.63	− 0.079	0.99
	Fish consumption per week	33.33	− 10.23 to 76.90	0.25	0.13
	Years working with mercury	− 1.03	− 3.53 to 1.46	− 0.16	0.40
	Smelting amalgam to recovery gold	− 18.7	− 76.07 to 38.63	− 0.12	0.51
	Handling mercury to extract gold from ore, but no smelting	− 10.74	− 57.02 to 35.55	− 0.081	0.64
45	***mg NBMI/Kg/Day***^***2***^	***− 15.32***	***− 27.37 to − 3.27***	***− 0.41***	***0.014***
45	***mg NBMI/Kg/Day***	***− 19.60***	***− 31.41 to − 7.79***	***− 0.53***	***0.0021***
	Age in years	36.48	− 28.78 to 101.74	6.46	0.26
	Date of birth in years	35.93	− 29.7 to 101.6	6.32	0.27
	Fish consumption per week	***42.27***	***5.82 to 78.71***	***0.34***	***0.025***
	***Years working with mercury***	***− 2.53***	***− 4.60 to − 0.46***	***− 0.42***	***0.019***
	Smelting amalgam to recovery gold	− 23.50	− 72.35 to 25.35	− 0.15	0.33
	Handling mercury to extract gold from ore, but no smelting	− 16.74	− 54.90 to 21.42	− 0.14	0.38

***Bold-italicized*** results are statistically significant

All study subjects received 14 days of NBMI therapy. *Kg* kilogram, *mg* milligram, *NBMI* N,N′bis-(2-mercaptoethyl) isophthalamide

Linear regression revealed on study day 15 (after study subjects received 14 days of therapy), on a mg NBMI/Kg bodyweight/day basis, urinary Hg concentrations significantly decreased by about 18 µg Hg/L. After adjustment for the covariates examined, on a mg NBMI/Kg bodyweight/day basis, urinary Hg concentrations significantly decreased, slightly more than without adjustment, by about 20 µg Hg/L. On study day 45 (30 days after study subjects received the last day of therapy), on a mg NBMI/Kg bodyweight/day basis, urinary Hg concentrations significantly decreased by about 15 µg Hg/L in the unadjusted model and by about 20 µg Hg/L in the adjusted model. Similar results were observed using the Spearman rank correlation test statistic. There was a significant inverse relationship between urinary Hg concentrations and daily doses of NBMI on study days 15 (ρ = − 0.49) and 45 (ρ = − 0.35).

**Fig. 2:**
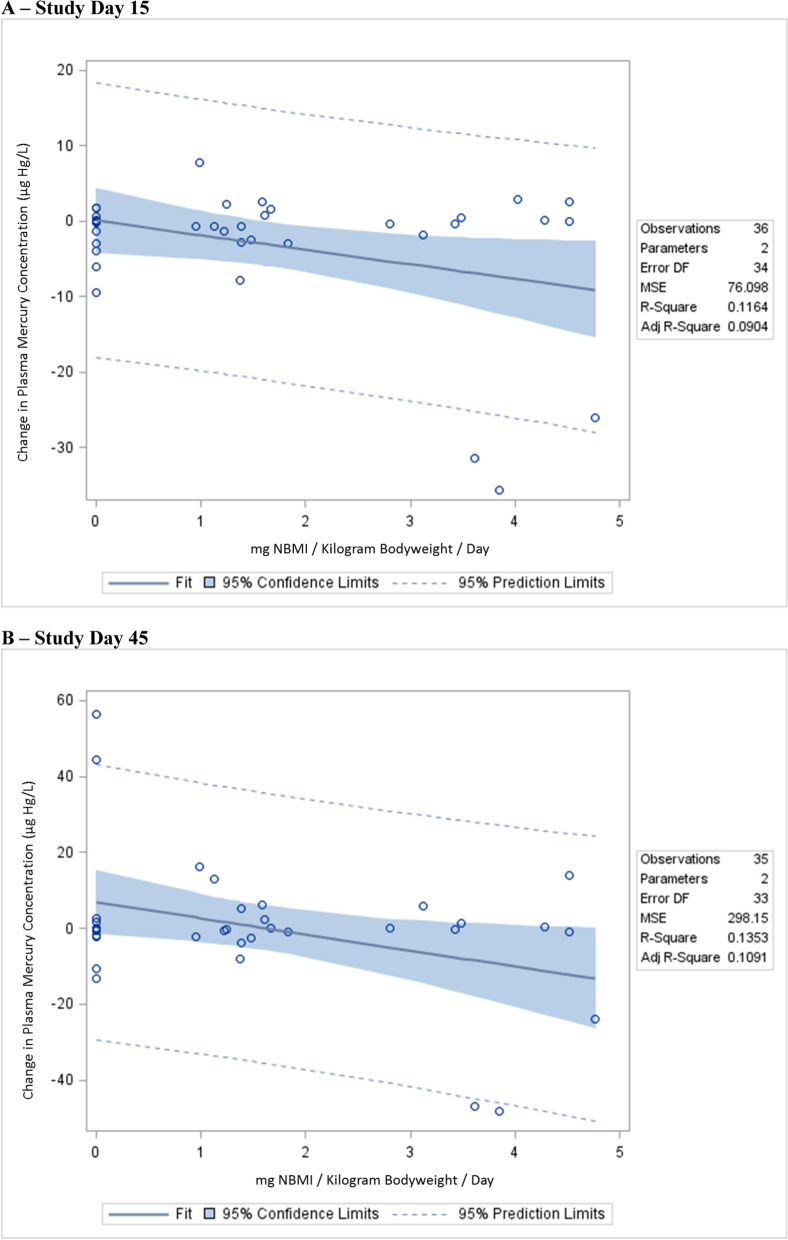
A summary of the correlation between plasma mercury concentrations and daily doses of NBMI.  **A** study day 15,  **B** study day 45

 Table 3; Fig. 2 show the correlation observed between changes in plasma Hg concentrations with NBMI therapy for all study subjects.

**Table 3:** A summary of the linear regression correlation between plasma mercury concentrations and daily doses of NBMI

Study day	Variable	β-coefficient	95% Confidence interval	Standardized estimate	p-value
15	***mg NBMI/Kg/Day***	***− 1.93***	***− 3.76 to − 0.077***	***− 0.34***	***0.042***
15	***mg NBMI/Kg/Day***	***− 2.32***	***− 4.52 to − 0.13***	***− 0.41***	***0.039***
	Age in years	− 0.83	− 13.02 to 11.39	− 0.94	0.89
	Date of birth in years	− 0.95	− 13.22 to 11.32	− 1.07	0.88
	Fish consumption per week	3.04	− 3.71 to 9.80	0.16	0.36
	Years working with mercury	− 0.24	− 0.62 to 0.15	− 0.25	0.22
	Smelting amalgam to recovery gold	1.05	− 7.84 to 9.95	0.046	0.81
	Handling mercury to extract gold from ore, but no smelting	− 0.73	− 7.91 to 6.44	− 0.039	0.84
45	***mg NBMI/Kg/Day***	***− 4.23***	***− 8.01 to − 0.44***	***− 0.37***	***0.030***
45	***mg NBMI/Kg/Day***	***4.32***	***− 8.31 to − 0.33***	***− 0.38***	***0.035***
	Age in years	13.96	− 8.09 to 36.02	8.03	0.21
	Date of birth in years	14.07	− 8.12 to 36.26	8.03	0.20
	***Fish consumption per week***	***14.62***	***2.30 to 26.94***	***0.38***	***0.022***
	Years working with mercury	− 0.52	− 1.22 to 0.18	− 0.28	0.14
	Smelting amalgam to recovery gold	7.41	− 9.10 to 23.92	0.15	0.37
	***Handling mercury to extract gold from ore, but no smelting***	***5.16***	***− 7.74 to 18.06***	***0.14***	***0.042***

***Bold-italicized*** results are statistically significant. All study subjects received 14 days of NBMI therapy

*Kg* kilogram, *mg* milligram, *NBMI* N,N′bis-(2-mercaptoethyl) isophthalamide

Linear regression revealed that on study day 15 (after study subjects received 14 days of therapy), on a mg NBMI/Kg bodyweight/day basis, plasma Hg concentrations significantly decreased by about 2 µg Hg/L in the unadjusted and adjusted models. On study day 45 (30 days after study subjects received the last day of therapy), on a mg NBMI/Kg bodyweight/day basis, plasma Hg concentrations significantly decreased by about 4 µg Hg/L in the unadjusted and adjusted models. By contrast the Spearman rank correlation test statistic failed to reveal significant inverse relationships between plasma Hg concentrations and daily doses of NBMI on study days 15 and 45.

**Fig. 3:**
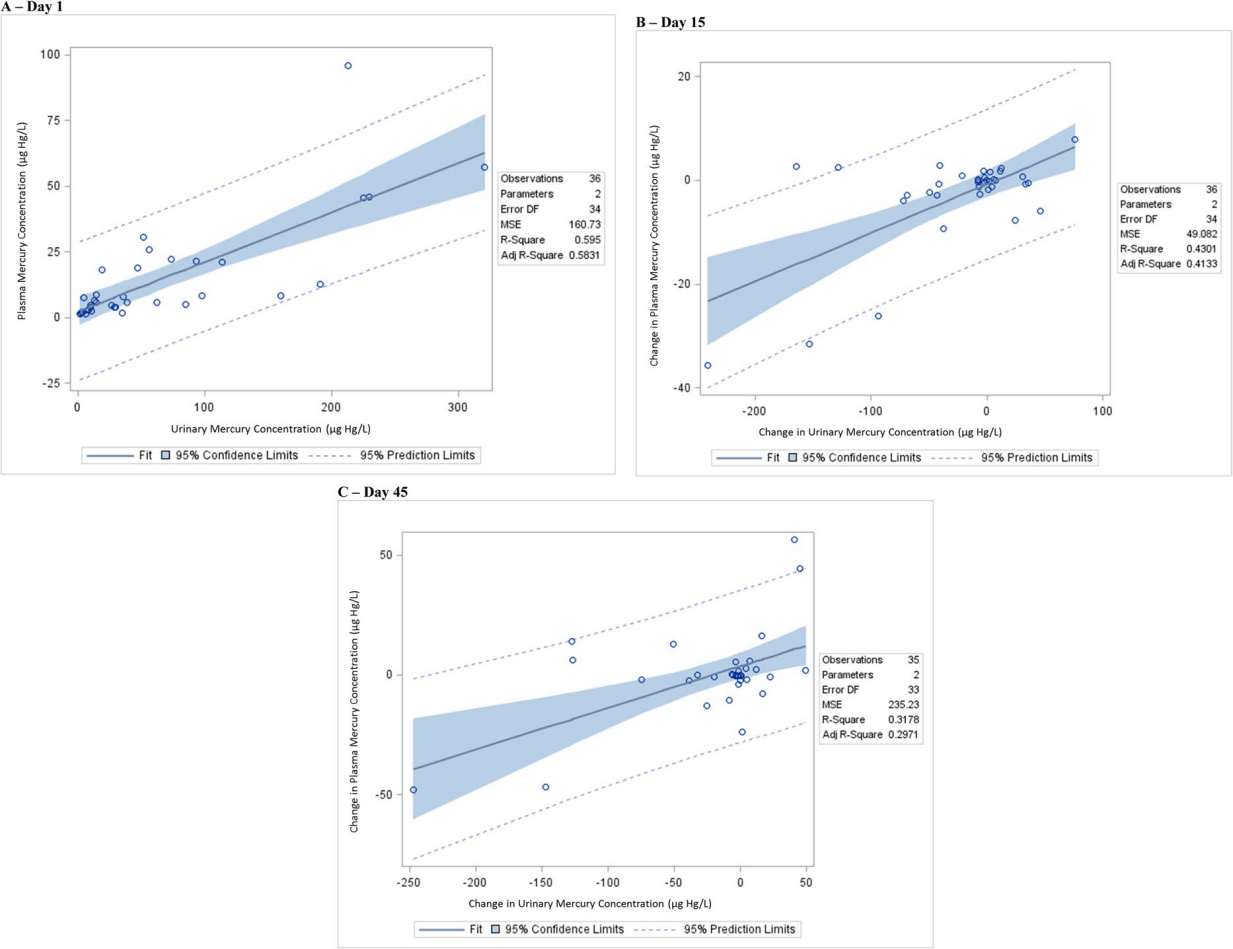
A summary of the correlation between the plasma mercury concentrations and the urinary mercury concentrations.  **A** day 1,  **B** day 15,  **C** day 45

 Table 4; Fig. 3 display the correlations between the concentrations of Hg in the plasma and urinary.

**Table 4:** A summary of the linear regression correlation between the plasma mercury concentrations and the urinary mercury concentrations

Study day	Variable	β-coefficient	95% Confidence interval	Standardized estimate	p-value
1	***Urinary mercury concentration***^***a***^	***0.19***	***0.14 to 0.24***	***0.77***	***< 0.0001***
15	***Change in urinary mercury concentration***^***b***^	***0.094***	***0.056 to 0.13***	***0.66***	***< 0.0001***
45	***Change in urinary mercury concentration***^***c***^	***0.17***	***0.084 to 0.26***	***0.56***	***0.0004***

***Bold-italicized*** results are statistically significant. All study subjects received 14 days of NBMI therapy

*Kg* kilogram, *mg* milligram, *NBMI* N,N′bis-(2-mercaptoethyl) isophthalamide

^a^The Spearman rank correlation test statistic yielded a significant direct relationship between urinary and plasma mercury concentrations (ρ = 0.80, 95% confidence interval = 0.64 to 0.89, p-value < 0.0001)

^b^The Spearman rank correlation test statistic was not significant (ρ = 0.27, 95% confidence interval = − 0.071 to 0.1954, p-value = 0.12)

^c^The Spearman rank correlation test statistic was not significant (ρ = 0.28, 95% confidence interval = − 0.066 to 0.56, p-value = 0.11)

On study day 1 (pretreatment) the concentration of Hg in the urine significantly correlated with the concentration of Hg in the plasma using linear regression. For every 1 µg Hg/L increase in urinary Hg concentration, the plasma Hg concentration increased by about 0.2 µg Hg/L. Similarly, it was observed following 14 days of therapy, changes in Hg concentration in the plasma and urine significantly correlated with each other on study days 15 and 45.

## Discussion

The present study provides new and compelling perspectives regarding the dose-dependent effectiveness of NBMI therapy to reduce Hg intoxication in an adult male population exposed to significant amounts of Hg. The results of this study showed there were dose-dependent effects of NBMI therapy to significantly lower plasma and urine Hg concentrations. The results also showed that covariates examining various types of environmental Hg exposure did not impact the effectiveness of NBMI therapy to reduce plasma and urine Hg concentrations.

The results observed in this study confirm and extended those reported originally from the randomized, placebo-controlled trial (Schutzmeier et al. 2018). In the original study, statistical models were constructed to evaluate different categorical NBMI treatment groups (placebo, 100 mg NBMI, and 300 mg NBMI) for changes in the Hg concentrations (continuous variable) present in plasma and urine samples collected on study days 15 and 45 as compared to samples collected on study day 1. Unfortunately, unlike appropriately constructed clinical categorical models where *a priori* there is general consensus as to the expected effects in different categorical groups (Fuller et al. 2016), the investigators were hampered by limited knowledge as to the potential therapeutic effectiveness of NBMI therapy in humans (i.e., they did not *a priori* know, what, if any, effectiveness different NBMI doses would have on reducing Hg concentrations in plasma and urine samples). The aforementioned limitation was further compounded by the fact that each NBMI treatment group was of relative small size (about 12 study subjects). The small size of each NBMI treatment group made them subject to the impacts of potential data outliers and reducing the statistical power of the study. Despite, these limitations, the investigators did observed when comparing the 300 mg NBMI treatment group to the placebo group that urinary Hg concentrations were significantly decreased for samples collected on study day 15 as compared to samples collected on study day 1, but they did not observe other significant effects.

By contrast, the present study utilized linear regression statistical models to evaluate for all 36 study subjects their NBMI treatment status as continuous variable (mg NBMI/Kg bodyweight/day) and their changes in Hg concentrations (continuous variable) present in plasma and urine samples collected on study days 15 and 45 as compared to samples collected on study day 1. The use of linear regression statistical models is appropriate, in this study because they allowed for determination of how hypothesized variable(s) will predict an outcome (i.e., it was hypothesized that NBMI therapy would potentially significantly reduce Hg concentrations in urine and plasma, but the effective dose of NBMI therapy was *a priori* unknown), define how those variables are to be constructed, specify a statistical framework within which the predictor variable(s) interact, and compute fixed coefficient values for each predictor variable (Fuller et al. 2016). Thus, a final linear regression statistical model provides a formula from which the likelihood or magnitude of an outcome variable can be estimated given the presence of selected predictor variables and it can be used to predict the outcome in other populations (Fuller et al. 2016).

In this study, β-coefficients (with 95% confidence intervals) were generated from the linear regression statistical models constructed to evaluate the potential dose-dependent effectiveness of NBMI therapy. The β-coefficients are the slope of the trend lines generated to describe the overall interrelatedness of the data points in each of our analyses. The 95% confidence intervals for each β-coefficient are the range, in which, with 95% certainty each β-coefficients lies. The β-coefficients calculated revealed that 14 days of NBMI therapy on a mg NBMI/Kg bodyweight/day basis (study day 15) significantly decreased urinary Hg concentrations by 18–20 µg Hg/L and plasma Hg concentrations by about 2 µg Hg/L. The β-coefficients also reveal, 30 days after 14 days of NBMI therapy on a mg NBMI/Kg bodyweight/day basis (study day 45), significantly decreased Hg concentrations in the plasma by 4 µg Hg/L and urine by 15–20 µg Hg/L.

It is hypothesized that initial reductions in the plasma and urine concentrations of Hg observed on study 15 reflect the ability of NBMI therapy to rapidly increase systemic NBMI concentrations allowing Hg-NBMI complexes to form for the more easily accessible and biologically active forms of Hg in the body. It is hypothesized the absence of rebound in plasma and urine Hg concentrations observed on study day 45, supports, not only that NBMI therapy was able to form Hg-NBMI complexes with more easily accessible and biologically active forms of Hg in the body, but that NBMI was able to significantly penetrate into tissues and organs throughout the whole-body and form Hg-NBMI complexes. Rebound in heavy metal concentrations following short courses of chelation therapy is a well-known phenomenon, which, was described as the redistribution of heavy metals from regions of the body less accessible to chelating agents to those more easily accessible (Mann and Travers 1991). The improved efficacy of NBMI therapy relative to other chelating agents is supported by its design as a lipophilic chelating agent with enhanced Hg binding capabilities.

In biological support of the observations made in this study regarding effectives of NBMI therapy for Hg intoxication, it was published that NBMI was able to form stable and biologically inactive complexes with Hg under physiological conditions (Zaman et al. 2007). In addition, studies in rats revealed that after the oral administration of NBMI, the half-life of NBMI in the plasma was 6.18 h (Clarke et al. 2012). Peak concentrations of NBMI were observed at 2 h post-ingestion in the brain, kidney, liver, spleen, bone marrow, small intestine, and subcutaneous fat. The amount of NBMI remaining after 24 h was about 13.4% of that retained after 2 h. These observations reveal oral administration of NBMI can significantly increase blood and tissue NBMI concentrations within hours of administration, and that blood and tissue NBMI concentrations of NBMI slowly decrease overtime. These previous observations support the ability of NBMI to penetrate tissues/organs with elevated Hg concentrations and form Hg-NBMI complexes that are non-toxic (Clarke et al. 2012).

There are several animal model systems that reveal the effectiveness of NBMI therapy to reduce Hg intoxication. For example, investigators examined the effect of NBMI therapy on preventing acute mercuric chloride (HgCl_2_) toxicity in rats (Clarke et al. 2012). These investigators observed, when comparing young rats treated with HgCl_2_ and NBMI, as compared to Hg alone, there was a significant increase in survivability. It was observed that Hg doses resulting in the death of 100% of the young rats within 7 days (1 mg HgCl_2_/Kg) or within 2 days (2 mg HgCl_2_/Kg), when administered a subcutaneous injection of NBMI within 20–25 min, 100% of the young rats were alive at 7 days. Other investigators evaluated the ability of NBMI therapy to prevent acute methyl-Hg intoxication in *Caenorhabditis elegans* (*C. elegans*) (Ke et al. 2020). These investigators observed that co-treatment of NBMI with methyl-Hg exposure significantly reduced in the worms the death rate, structural damage in neurons, and restored antioxidant response levels.

It is also important to consider the effectiveness of NBMI therapy in light of its safety profile. In Sprague Dawley rats, injected up to 767 mg NBMI/Kg bodyweight over a 10 day period, there were no signs of weight loss or toxicity (ataxia, appetite suppression, diarrhea, or tremors) (Clarke et al. 2012). Oral administration of NBMI to Wistar rats was undertaken to determine the LD-50. It was observed that the LD-50 was in excess of 5 g NBMI/Kg bodyweight (no deaths or other signs of toxicity with the exception of some diarrhea was observed at 5 g NBMI/Kg bodyweight, and the diarrhea observed was attributed to the doses of corn oil needed to deliver such a large amount of NBMI) (Clarke et al. 2012). Twenty-eight day toxicity studies in male and female Wistar rats revealed that doses as high as 1 g NBMI/Kg bodyweight per day (administered for 28 days) revealed by microscopic and histological analyses no changes indicative of toxicity in the organs evaluated (with the exception of some mild to moderate B-cell proliferation in the spleens). Further, NBMI therapy was observed to produce no abnormalities in the blood concentrations of calcium, chloride, magnesium, potassium, sodium, and phosphorus. Also, no adverse effects were observed on red blood cell count, hematocrit, and hemoglobin levels and there were no indications of low blood zinc or copper levels. The human randomized, placebo-controlled NBMI trial revealed equally reassuring safety regarding NBMI therapy in humans (Schutzmeier et al. 2018). No serious adverse events occurred in the clinical trial and no one needed to discontinue the trial due to adverse events. The majority of the adverse events observed in the clinical trial were headaches and gastrointestinal events that occurred evenly between the NBMI treatment and placebo groups. All told, these studies in animals and humans help to establish a robust safety profile for NBMI therapy.

### Study limitations

There are several important potential study limitations to consider regarding the present study.

First, this study was not able to estimate the exact Hg dose received by each study subject. The study subjects examined were selected from a mining region of Ecuador with known Hg exposure. Further, the study subjects were also selected because each had a lab test revealing elevated urinary Hg concentrations (> 15 µg Hg/L). The present study evaluated the potential impact of environmental Hg exposures on NBMI effectiveness by including covariates in the statistical models constructed based upon each study subject’s initial questionnaire answers regarding environmental Hg exposures. It is recommended that future NBMI treatment clinical trials be conducted on study populations with continuous and estimable amounts of Hg exposure. One such population might by those with elevated Hg vapor exposure from dental amalgams (Geier and Geier 2022).

Second, only limited dosing regimens of NMBI therapy were utilized in the clinical trial (100 mg or 300 mg per day). As a result, the ability of this study to explore the potential benefits of higher NBMI dosing (> 5 mg NBMI/Kg bodyweight/day) was limited. Since, the present study observed a significant dose-dependency between increasing mg NBMI/Kg bodyweight/day and reductions in Hg concentrations in plasma and urine; it is presumed that higher NBMI doses may produce even greater reduction in plasma and urine Hg concentrations. In light of NBMI’s safety profile and its ability to dose-dependently reduce urinary and plasma Hg concentrations, it is recommended that future studies examine higher daily doses of NBMI (e.g., 600 mg per day).

**Fig. 4:**
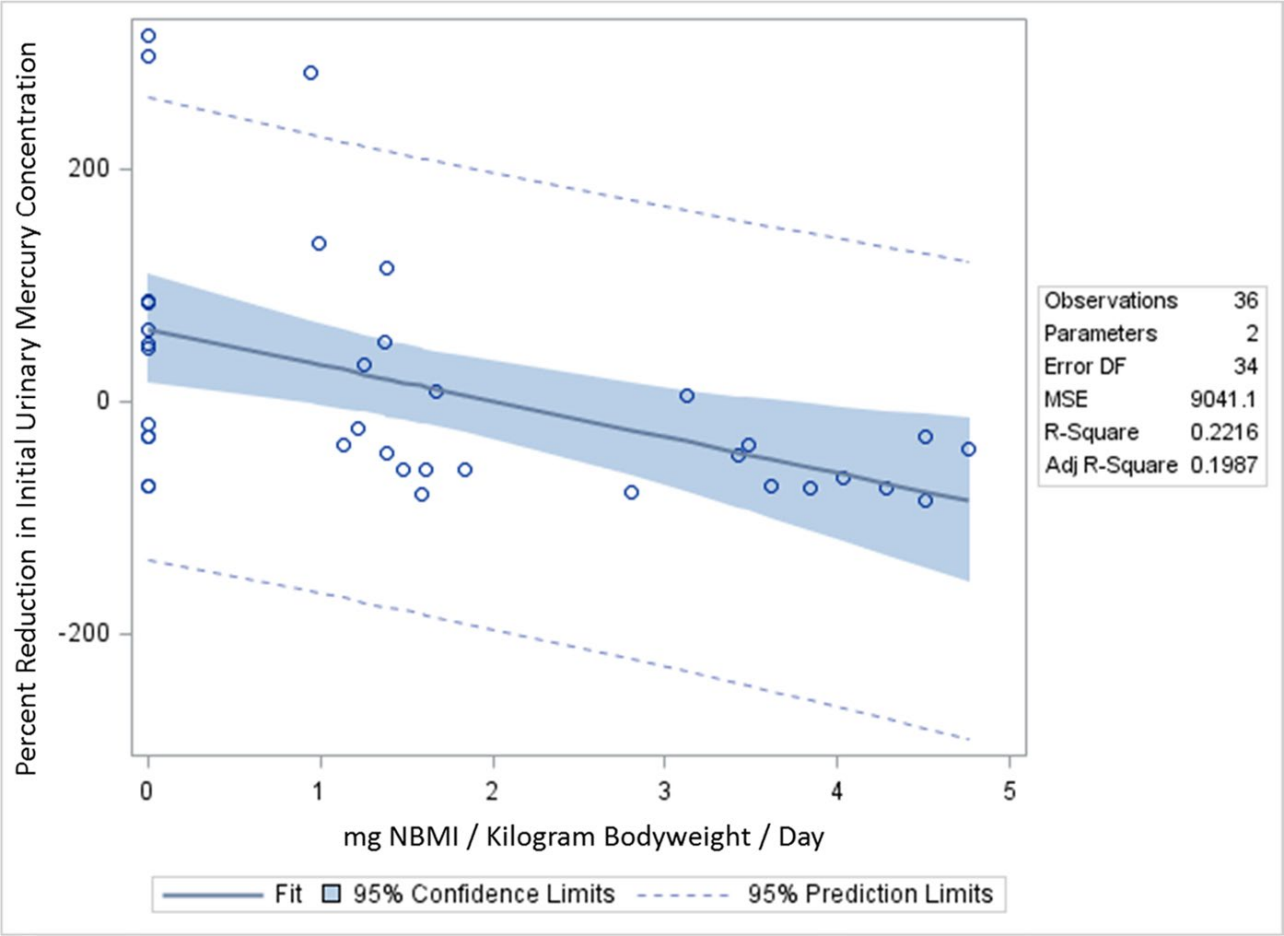
A summary of the percent reduction in initial urinary mercury concentration on study day 15

Third, measured changes in plasma and urine Hg concentrations as compared to baseline were examined. This method of comparison weights those persons’ having the largest numeric change with the greatest weight and those with the smallest numeric change with the least weight. It is possible that other methods of considering the data may yield different results. For example, another method of data analysis would be to evaluate the percentage reduction in Hg concentrations as compared to the initial measurement. This would equally weight those with large and small numeric changes. As shown in Table 5; Fig. 4, we undertook such an analysis for urinary Hg concentrations collected on study day 15 as compared to study day 1.

**Table 5:** A summary of the linear regression correlation between the percent reduction in urinary mercury concentrations and daily doses of NBMI

Study day	Variable	β-coefficient	95% Confidence interval	Standardized estimate	p-value
15	***mg NBMI/Kg/Day***	***− 30.86***	***− 51.03 to − 10.70***	***− 0.47***	***0.0038***
15	***mg NBMI/Kg/Day***	***− 29.63***	***− 53.67 to − 5.60***	***− 0.45***	***0.018***
	Age in years	− 3.81	− 137.2 to 129.5	− 0.37	0.95
	Date of birth in years	− 2.24	− 136.4 to 131.9	− 0.22	0.97
	Fish consumption per week	35.03	− 38.83 to 108.9	0.16	0.34
	Years working with mercury	− 0.92	− 5.15 to 3.32	− 0.084	0.66
	Smelting amalgam to recovery gold	− 5.39	− 102.6 to 91.8	− 0.020	0.91
	Handling mercury to extract gold from ore, but no smelting	− 16.27	− 94.74 to 62.19	− 0.075	0.67

***Bold-italicized*** results are statistically significant. All study subjects received 14 days of NBMI therapy

*Kg* kilogram, *mg* milligram, *NBMI* N,N′bis-(2-mercaptoethyl) isophthalamide

This analysis using linear regression revealed, on a mg NBMI/Kg bodyweight/day basis, there was a significant 30% reduction in urinary Hg concentrations. It would be worthwhile in future studies to explore other methods of measuring changes in urinary and plasma Hg concentrations with NBMI therapy.

Fourth, this study examined measured total Hg concentrations in plasma and urine on specific study days. It is possible that other samples such as feces or tissues or measurements at different time points may show different results. In addition, no Hg speciation (inorganic or organic) was undertaken on the plasma and urine samples examined. It is possible that different Hg species may be differentially impacted by NBMI therapy. It is recommended that future studies examine the impact of NBMI therapy on other types of samples collected at different time points while considering Hg speciation.

Fifth, the present study used linear regression statistical modeling, which requires five key assumptions: linear relationship, multivariate normality, no/little multicollinearity, no auto-correlation, and homscedasticity. Linear regression statistical modeling in SAS provides diagnostic plots to evaluate the fit of the model, to determine whether the data satisfy various assumptions, and to identify outliers and high-leverage points. Overall, the diagnostic plots generated for the data examined in this study generally supported the use of linear regression statistical modeling, but it was also determined that the urinary Hg concentration data was more robust than the plasma Hg concentration data.

In order to evaluate the impact of other correlation statistics on the results observed in this study, additional analyses were undertaken to examine the correlation between NBMI therapy and urinary and plasma Hg concentrations using the Spearman rank statistic in SAS. The assumptions of the Spearman rank statistic are different for those from the linear regression statistic, and include: random sample, monotonic association exists between 2 variables, and the variables are at least ordinal (ratio, interval, continuous (no nominal data)). The Spearman rank statistic is also less sensitive than the linear regression to outlier values. Consistent with the results observed from linear regression analyses, Spearman rank correlation analyses revealed that NBMI therapy dose-dependently reduced urinary Hg concentrations on study days 15 (ρ = − 0.49, 95% confidence interval = − 0.70 to − 0.19, p-value = 0.0021) and 45 (ρ = − 0.35, 95% confidence interval = − 0.61 to − 0.015, p-value = 0.038). By contrast, Spearman rank correlation analyses did not yield consistent results with linear regression analyses on the dose-dependent ability of NBMI therapy to reduce Hg concentrations in the plasma on study days 15 and 45. In addition, the Spearman rank correlation test statistic yielded a significant inverse dose-dependent relationship between the percent reduction in urinary Hg concentrations on day 15 and NBMI therapy (ρ = − 0.573, 95% confidence interval = − 0.75 to − 0.29, p-value = 0.002). A potential explanation for the divergence in results for plasma Hg concentrations may stem from how outlier values were considered in the statistic models utilized in this study. Given the current state of limited knowledge as to the effectiveness of NBMI therapy, we believe it would be inappropriate to exclude/minimize any values from the statistical models constructed in this study. All told, despite some differences in statistically significant results between the linear regression and Spearman rank correlation analyses, it is believed that overlap in results, particularly for urinary Hg concentrations, and consistency in the direction of the results (even if they were not statistically significant in both models), collectively point to the effectiveness of NBMI therapy to significantly lower Hg body-burden. It is recommended that future studies consider other statistical modeling techniques to evaluate NBMI effectiveness.

## Conclusion

The present post hoc analysis study utilizing novel analytical methods provides important insights into the effectiveness of NBMI therapy to reduce Hg intoxication. It was observed that daily NBMI therapy over 14 days in a randomized and controlled clinical trial among an Ecuadorian miner population with confirmed elevated Hg concentrations dose-dependently reduced urinary and plasma Hg concentrations. These reductions were observed at the end of NBMI therapy and persisted 30 days after cessation of NBMI therapy. Given the robust safety profile of NBMI in humans and animals, and the results of this study strongly supporting the ability of NBMI therapy to treat Hg intoxication, we believe continued and expanded study of this important compound are warranted, especially in light of the fact that there are many global populations at significant risk for suffering Hg intoxication.
